# Diagnosis of bacteremia in pediatric oncologic patients by in-house real-time PCR

**DOI:** 10.1186/s12879-015-1033-6

**Published:** 2015-07-23

**Authors:** Milene Gonçalves Quiles, Liana Carballo Menezes, Karen de Castro Bauab, Elke Kreuscher Gumpl, Talita Trevizani Rocchetti, Flavia Silva Palomo, Fabianne Carlesse, Antonio Carlos Campos Pignatari

**Affiliations:** Special Laboratory of Clinical Microbiology (LEMC), Federal University of São Paulo/UNIFESP, São Paulo, Brazil; Institute of Pediatric Oncology IOP-GRAACC, Federal University of São Paulo, São Paulo, Brazil

**Keywords:** Real time PCR, Resistance genes, Bloodstream infections, Bacterial pathogens

## Abstract

**Background:**

Infections are the major cause of morbidity and mortality in children with cancer. Gaining a favorable prognosis for these patients depends on selecting the appropriate therapy, which in turn depends on rapid and accurate microbiological diagnosis. This study employed real-time PCR (qPCR) to identify the main pathogens causing bloodstream infection (BSI) in patients treated at the Pediatric Oncology Institute IOP-GRAACC-UNIFESP-Brazil. Antimicrobial resistance genes were also investigated using this methodology.

**Methods:**

A total of 248 samples from BACTEC® blood culture bottles and 99 whole-blood samples collected in tubes containing EDTA K2 Gel were isolated from 137 patients. All samples were screened by specific Gram probes for multiplex qPCR. Seventeen sequences were evaluated using gender-specific TaqMan probes and the resistance genes *bla*_SHV_, *bla*_TEM_, *bla*_CTX_, *bla*_KPC_, *bla*_IMP_, *bla*_SPM_, *bla*_VIM_, *van*A, *van*B and *mec*A were detected using the SYBR Green method.

**Results:**

Positive qPCR results were obtained in 112 of the blood culture bottles (112/124), and 90 % agreement was observed between phenotypic and molecular microbial detection methods. For bacterial and fungal identification, the performance test showed: sensitivity 87 %; specificity 91 %; NPV 90 %; PPV 89 % and accuracy of 89 % when compared with the phenotypic method. The *mec*A gene was detected in 37 samples, extended-spectrum β-lactamases were detected in six samples and metallo-β-lactamase coding genes in four samples, with 60 % concordance between the two methods. The qPCR on whole blood detected eight samples possessing the *mec*A gene and one sample harboring the *van*B gene. The *bla*_KPC_, *bla*_VIM_, *bla*_IMP_ and *bla*_SHV_ genes were not detected in this study.

**Conclusion:**

Real-time PCR is a useful tool in the early identification of pathogens and antimicrobial resistance genes from bloodstream infections of pediatric oncologic patients.

## Background

In recent decades, the successful treatment of malignancies in children and adolescents has been increasing, with up to 70–80 % of cases having a favorable outcome. However, immunosuppression resulting from treatment makes these patients more susceptible to infections, which are the leading cause of death in this population [1, 2].

In these patients, bacteremia often results from infection with one or more microorganisms, predominantly members of the Enterobacteriaceae, *Pseudomonas aeruginosa*, *Streptococcus* and *Staphylococcus* [3, 4]. In a Brazilian study of 46 patients who had undergone chemotherapy, 26 were neutropenic and 53 % had positive blood cultures (BC) [1]. Another similar study obtained positive BCs from 41 hospitalized children among a total of 110 patients tested [4]. In Italy, an analysis at 18 centers of 176 episodes of infection found that 123 (64 %) of these cases occurred in neutropenic children versus 68 (36 %) in non-neutropenic children [5].

Bacterial isolation using conventional microbiological techniques rarely exceeds 25 % in children with clinical signs and laboratory findings suggestive of invasive bacterial infection [6, 7]. It is also worth noting that in 40 % of children that have a clinical diagnosis of sepsis and 75 % of high-risk febrile neutropenia cases, the microorganism cannot be detected by currently available conventional microbiological techniques [8].

In this context, the aim of this study was to evaluate the performance of in-house real time PCR (qPCR), applied directly to BACTEC® bottles and whole blood samples, for the detection of 17 relevant microorganisms and 10 antimicrobial resistance genes causing bacteremia in children with cancer. Our hypothesis was that this molecular method could be an accurate and sensitive method for early bacteremia diagnosis in this population.

## Methods

### Patients

We designed a prospective study that was conducted between October 2010 and June 2012 and involved 137 patients admitted to the Institute of Pediatric Oncology (IOP-GRAACC, Brazil), which supports children and adolescents with cancer. Informed consent was obtained from patients, and the study was approved by the Clinical Research Ethics Committee of the hospital.

### Blood collection and phenotypic bacterial identification

Blood samples were collected as part of the standard patient care procedures. The criteria for collecting blood cultures included: fever, defined as a temperature above 38 °C or above 37.5 °C for three consecutive measurements, or suggestive clinical signs of sepsis.

At the onset of fever or in the presence of clinical symptoms, two sets of BCs were taken from peripheral venous access or from the central line catheter. Bloodstream infection (BSI) was defined as the isolation of a bacterial or fungal pathogen from at least one BC [9]. All episodes of BSI were then sub-classified as a single-agent (Gram-positive, Gram-negative or fungi) or polymicrobial. For polymicrobial BSI, two or more pathogens were isolated from a single BC or at least two separate BCs, 96 h apart. BCs were performed using the BACTEC® 9240 instrument (Becton Dickinson, Microbiology Systems, Cockeysville, MD, USA). BC bottles were designated positive after detection of bacterial growth. Samples were negative when no bacterial growth was detected after five days incubation. Identification at the species level and susceptibility testing were performed using the automated system Phoenix 100 (Becton Dickinson, Microbiology Systems).

Whole blood samples were collected at the same time and following the same criteria when possible. These samples were not analyzed phenotypically but instead were subjected to molecular assays.

### Molecular detection of microorganisms and resistance genes

Chromosomal DNA was extracted from 500 μl of positive and negative BC samples after incubation on BACTEC® 9240, using the phenol–chloroform method (Brazol; LGC Biotechnologia, Cotia, Brazil) with 300 mg glass beads (0.3 mm diameter; Scientific Industries, Bohemia, NY, USA), and were processed in a disruptor Genie (Scientific Industries) for 10 min to achieve cell lysis. In parallel, chromosomal DNA was extracted from 1.5 ml of whole blood samples using the QIAamp DNA blood kit (Qiagen Inc., Valencia, CA, USA). Extracted DNA was diluted (1:10) using DNase/RNase-free distilled water UltraPure (Invitrogen, Carlsbad, CA, USA) for reducing the inhibitor reagents in the sample. Extracted DNA was used for the detection of pathogens and resistance genes by our in-house qPCR assay.

The primers used for detection of the resistance genes *bla*_SHV_, *bla*_TEM_, *bla*_CTX-M_, *bla*_IMP_, *bla*_SPM_, *bla*_VIM_, *bla*_KPC_, *van*A, *van*B and *mec*A, have been described previously [8, 10–12], as have the primers and TaqMan probes for multiplex qPCR detection of Gram-positive (GP) and Gram-negative (GN) bacteria and specific-species detection of *Enterococcus* spp., coagulase negative *Staphylococcus* (CoNS), *S. aureus*, *Streptococcus pneumoniae*, *Escherichia coli*, *Klebsiella pneumoniae*, *Enterobacter cloacae*, *Proteus mirabilis*, *Salmonella* spp., *Serratia marcescens*, *Acinetobacter baumannii*, *Pseudomonas aeruginosa*, *Stenotrophomonas maltophilia*, *Mycobacterium tuberculosis*, *Aspergillus* spp., *Candida* spp. and *Fusarium* spp. [13, 14]. A primer set for the hemochromatosis gene (HFE) was designed to be used as an internal control of DNA extraction. The analytical validations of these tests have been described previously [14].

The presence of bacterial DNA in the sample was first screened by a TaqMan-based multiplex qPCR using universal primers for the 16S rDNA gene for differentiation between Gram-positive and Gram-negative bacteria. Species identification was performed by a Taqman-based monoplex qPCR. Amplification for the TaqMan probe reaction was performed in a 20 μl reaction volume, using 10 μl TaqMan Universal Master Mix 2× (Applied Biosystems), 5 μl template DNA, 0.5 μM each primer and 0.3 μM probe. The qPCR conditions were as follows: 50 °C for 2 min and 95 °C for 10 min; 40 cycles of 95 °C for 15 s and 60 °C for 60 s.

Resistance gene amplification by SYBR Green monoplex qPCR was performed in a 25 μl reaction mixture containing 12.5 μl Platinum SYBR Green qPCR SuperMix (Invitrogen, Carlsbad, CA, USA), 0.5 μM each primer, and 5 μl of DNA extracted from samples. The qPCR conditions were as follows: 50 °C for 2 min and 95 °C for 10 min; 40 cycles of 95 °C for 15 s and 60 °C for 60 s; and a melting curve step (from 68 °C to 95 °C in increments of 0.5 °C/s). Metallo-β-lactamase (MβL) detection was performed by multiplex qPCR [13]. The ABI 7500 real-time PCR System (Applied Biosystems) instrument was quantified online and at the endpoint with the sequence detection system software (version 2.0; Applied Biosystems).

The number of DNA molecules present in the sample was determined based on the amount of fluorescence detected by the qPCR instrument. This quantified fluorescence resulted in a Cycle Threshold value (Ct) that corresponds to the number of amplification cycles required to obtain a given amount of DNA [15].

### Additional tests to confirm the susceptibility profile

Samples with discordant susceptibility profiles between molecular and phenotypic analyses were subjected to further testing to confirm the results. For GN samples with discordant MβL or extended-spectrum β-lactamase (ESβL) profiles, we conducted a qPCR assay directly from the clinical strain recovered from our collection of microorganisms. These strains were stored at −20 °C in tryptone soya broth containing glycerol and were subcultured on blood agar for isolation. DNA extraction and qPCR were performed following the protocols described above.

For methicillin-resistant *Staphylococcus* strains with discordant profiles, we performed the qPCR directly from the clinical strain and confirmed the minimum inhibitory concentration (MIC) for oxacillin with an E-test®. The diameter of the zone for cefoxitin was determined by the disc diffusion test following the Clinical & Laboratory Standards Institute recommendations [16].

### Statistical analysis

Data collection and statistical analysis were performed using SPSS version 17.0 software (SPSS Inc., Chicago, IL, USA) and Microsoft Office Excel 2010 (Microsoft, Redmond, WA, USA). Comparison of the BC identification by phenotypic methods versus qPCR results was evaluated by *χ*2 tests. Discordance between the results from the two methods was assessed using McNemar’s test (statistical significance was set at a two-tailed exact test, based on a binomial distribution with *p* = q = 0.5). The κ statistic was calculated to measure the level of agreement between phenotypic and qPCR results directly from BC bottles.

## Results

A total of 347 samples from 137 patients admitted to the IOP/GRAACC were included in this study. These samples included 248 BC bottles and 99 whole blood samples collected in EDTA tubes. Clinical data from patients were collected by the Center of Infection Control at the Institute. Among these 248 BC bottles, 53.2 % (*n* = 132) were obtained from peripheral venous access and 46.8 % (*n* = 116) from the central line catheter.

The average age of patients was nine years old (ranging from 0 to 25); 64 (46 %) were female and 74 (54 %) were male. Underlying diseases were blastoma (19 %), acute lymphoblastic leukemia (15 %), general tumors (15 %), sarcoma (13 %) and non-Hodgkin lymphoma (12 %). Among 137 patients, 17 were submitted for allogeneic bone marrow transplant, 15 for autologous bone marrow transplant and one for liver transplantation. Sulfamethoxazole and trimethoprim antibiotic prophylaxis was used in patients with leukemia, lymphoma and in those with a solid tumor who received corticosteroids above 2 mg/kg.

### Phenotypic testing

Initial phenotypic analyses from 248 BCs identified 127 (51.2 %) negative and 121 (48.8 %) positive samples. Three episodes were classified as polymicrobial with two agents detected in one sample. Thus, a total of 124 strains were identified by this method: 67 (54.0 %) GP, 43 (34.7 %) GN species. The main pathogens identified were CoNS (*n* = 35) and *P. aeruginosa* (*n* = 14) (Table 1). Fungi species were detected in 14 (11.3 %) samples and the main agent identified was *Candida* spp. (*n* = 12).

**Table 1:** Comparison of phenotypic and molecular assays apllied to bloodculture bottles

	Phenotipyc detection and identification by phenotypic analisys (*n* = 124)	Molecucar detection by GP/GN multiplex PCR	Concordance (%)	Molecular identification by specific-species qPCR (*n* = 124)	Concordance (%)	Average Cycle threshold
*S. aureus*	12	12	100 %	10	83.40 %	17,683
SCoN	35	28	80 %	32	91.40 %	20,608
*Enterococcus* spp.	5	5	100 %	5	100 %	24,923
*S. pneumoniae*	1	1	100 %	1	100 %	20,977
*K. pneumoniae*	3	3	100 %	3	100 %	29,111
*E. cloacae*	6	6	100 %	6	100 %	20,919
*E. coli*	8	7	87.50 %	7	87.50 %	18,995
*S. marcescens*	2	2	100 %	2	100 %	25,252
*P. aeruginosa*	14	13	92.80 %	13	92.80 %	19,395
*S. malthofilia*	1	1	100 %	1	100 %	36,974
*Acinetobacter* spp.	1	1	100 %	1	100 %	27,993
*Candida* spp.	12	12	100 %	3	25 %	25,464
Other fungi	2	2	100 %	^a^	^a^	^a^
*Streptococcus* sp.	9	7	77.80 %	^a^	^a^	^a^
Others	13	12	92.30 %	^a^	^a^	^a^

^a^Species not included in the in-house qPCR standardized assay described in this study

The susceptibility testing performed on positive samples detected 31 (88 %) methicillin-resistant CoNS, 8 (66 %) methicillin-resistant *S. aureus* and two vancomycin-resistant *E. faecium*. Nine ESβL Enterobacteriaceae producers and 12 carbapenem-resistant *P. aeruginosa* strains were detected among the GN strains.

### Molecular testing from BC bottles

All samples included in the study were positive for the HFE gene, which indicated that the DNA had been successfully extracted. qPCR for GP/GN 16S rDNA from the 248 BCs samples detected 112 (90.3 %) positive samples versus the 124 detected by BC processing. Among 69 GP and 42 GN strains identified by BC, the qPCR method was able to identify 58 (86.5 %) GP and 40 (95.2 %) GN samples (Table 1). The overall concordance between the phenotypic and molecular methods was 89 % for GP and 90 % for GN detection with a Cohen κ coefficient of 0.775 (p-value < 0.001 and 95 % CI: 0.651–0.899).

Since the qPCR assay applied in this study was standardized to detect 17 different species from clinical samples, 24 samples containing different species were not identified. The species identified were 15 non-fermenter bacteria, 18 Enterobacteriaceae, 32 CoNS, 10 *S. aureus*, one Streptococci and five Enterococci (Table 1). There was a discrepancy for one sample that was identified as *S. aureus* by the phenotypic method and as CoNS by qPCR. The molecular method did not identify four GP samples. One false-positive sample was detected by qPCR with a 12,703 Ct rate, and this was identified as CoNS. *Candida* species were identified in 3 (25 %) samples.

For real-time PCR directly from BC bottles the performance test showed: sensitivity 90 %; specificity 88 %; negative predictive value (NPV) 88 %; positive predictive value (PPV) 90 % and accuracy of 89 % for GP and GN detection when compared with the phenotypic method from BC bottles. For bacterial and fungal identification the performance test showed: sensitivity 87 %; specificity 91 %; NPV 90 %; PPV 89 % and accuracy of 89 % when compared with the phenotypic method.

The qPCR for resistance genes detected four Enterobacteriaceae strains possessing the *bla*_CTX_ gene. Three of these samples were positive for the phenotypic detection of ESβL while one sample was ESβL negative and cephalosporin susceptible as determined by phenotypic analysis. In one ESβL-producer *K. pneumoniae* strain, the qPCR detected both *bla*_TEM_ and *bla*_CTX_ genes. One ESβL *E. coli* strain was identified that contained the *bla*_TEM_ gene. Four carbapenem-resistant *P. aeruginosa* were positive for the *bla*_SPM_ gene. Other GN resistant genes were not detected. The molecular method was not able to detect any resistance genes from eight carbapenem-resistant *P. aeruginosa* strains and six samples with an ESβL profile. The concordance between molecular and phenotypic analysis of the susceptibility profile was 60 %.

Among GP strains, 37 methicillin-resistant samples were detected that possessed the *mec*A gene (30 CoNS and seven *S. aureus*). There was a discrepancy for three samples. The concordance between both methods was 92.5 %. Two vancomycin-resistant Enterococci were positive for the *van*A gene with 100 % concordance.

Eight carbapenem-resistant *P. aeruginosa* isolates that tested negative for resistance genes on qPCR were subjected to qPCR directly from the clinical strain. Three of these samples were positive for *bla*_SPM_ in the second test. The other five isolates remained negative for resistance genes. One *E. coli* BC sample that tested positive for the *bla*_CTX_ gene and showed susceptibility to all cefalosporins was negative for this gene when tested directly from the isolate.

Three discordant *Staphylococcus* samples were subjected to confirmatory tests. Two methicillin-resistant strains (one CoNS and one *S. aureus*) that were negative for the *mec*A gene when tested from BC bottles, tested *mec*A positive in the qPCR directly from the isolated strain. One *S. aureus* sample considered phenotypically susceptible to methicillin tested positive for *mec*A both from BC bottles and the isolated strain. All three samples showed a high MIC for this drug and were resistant in the disc diffusion test.

### Molecular analysis of whole blood samples

A total of 99 whole blood samples were analyzed in this study. A positive result was obtained for the 16S rDNA qPCR in 12 (12.1 %) samples, of which two were amplified for both GP and GN and 10 only for GP (Table 2). The concordance between the phenotypic analysis of paired BCs of these samples and whole blood was 67.7 %.

**Table 2:** Phenotypic and molecular results from whole blood samples and paired BC bottles

BC Sample (BACTEC)	Whole blood sample (EDTA)	Specie isolated from BC bottle (Phoenix®)	Susceptibility profile (Phoenix®)	qPCR from BC bottles for resistance genes (ct)	qPCR from whole blood for resistance genes (ct)
195	176	*S. aureus*	Oxacillin S	mecA (21,855)	*mec*A (33,021)
178	117	^a^	^a^	ND	*mec*A (34,520)
190	152	*E. coli*	Carbapenem S	ND	ND
191	174	*P. aeruginosa*	Carbapenem S	ND	ND
283	133	*E. cloacae*	Carbapenem S	ND	ND
391	255	*P. putida*	Carbapenem S	ND	ND
404	232	*Enterococcus sp*	Vancomicyn S	ND	ND
341	233	*S. aureus*	Oxacillin R	*mec*A (28,550)	*mec*A (31,653)
371	240	*B. cepacia*	Carbapenem S	ND	ND
400	252	*B. cepacia*	Carbapenem S	ND	ND
477	288	*S. epidermidis*	Oxacillin R	*mec*A (23,914)	*mec*A (33,211)
389	237	*E. coli*	Carbapenem S	ND	ND
369	244	SCoN	Oxacillin S	ND	*mec*A (33,073)
372	247	*P. aeruginosa*	Carbapenem S	ND	ND
373	254	*S. epidermidis*	Oxacillin R	*mecA (15,683)*	*mec*A (29,909)
392	241	*S. haemolyticus*	Oxacillin R	*mecA (20,648)*	*mec*A (37,468)
431	276	SCoN	Oxacillin R	*mec*A (15,902)	*mec*A (37,320)
465	293	*P. aeruginosa*	Carbapenem R	ND	ND
441	273	*S. epidermidis*	Oxacillin R	ND	ND
422	264	*S. aureus*	Oxacillin R	*mec*A (16,508)	*mec*A (35,945)
423	265	SCoN	Oxacillin R	*mec*A (15,513)	*mec*A (36,217)
470	290	*E. faecium*	Vancomicyn R	*van*A (17,560)	*van*A (24,938)

*BC* Bloodculture bottle, *ct* cycle threshold, *R* resistant, *S* Susceptible, *ND* Not detected

^a^Negative sample at Phenotypic tests

The qPCR for specific species identified CoNS (*n* = 25), *S. aureus* (*n* = 2), *Enterococcus* spp. (*n* = 2), *S. pneumoniae* (*n* = 1), *E. coli* (*n* = 1), *P. aeruginosa* (*n* = 1) and *Candida* spp. (*n* = 1) with 55 % concordance with phenotypic identification from paired BCs. One sample showed a discrepancy with phenotypic detection of *E. cloacae* and qPCR detection of *S. aureus* and CoNS. We identified 14 false-positive samples from qPCR, one *Candida* spp. and 13 CoNS. This technique was not able to detect eight positive samples (8.1 %).

The *mec*A gene was identified from 11 samples of which three were methicillin-resistant on BC with 60 % concordance. The *van*A gene was detected in one vancomycin-resistant sample. Other resistance genes were not detected from whole blood samples. The degree of concordance between the phenotypic susceptibility test from paired BCs and qPCR from whole blood was 59 %.

The overall concordance between the qPCR from BC bottles and whole blood samples was 82 % with a Cohen K coefficient of 0.702 (*p*-value < 0.001 and 95 % CI: 0.502 e 0.903).

## Discussion

Patients with cancer, especially children, are susceptible to various infections because of immunosuppression caused by the treatment. These infections are difficult to treat and the agent is only identified in 22–39 % of cases [17]. Effective identification of the causative agent and the correct choice of antimicrobial therapy is crucial for reducing mortality in these patients [7].

When conventional techniques are used to identify bacteria and fungi, only up to 25 % of the episodes of bacteremia are detected in children with clinical and laboratory findings. In approximately 40 % of children diagnosed with sepsis and 75 % with neutropenia, the microorganism is not detected. Several studies have suggested that applying molecular methods directly to clinical samples could be a rapid and accurate tool for diagnosis in these patients [7]. In this study, we compared phenotypic and molecular assays for the detection and identification of microorganisms from clinical samples and performed susceptibility testing.

We observed that the molecular method was not able to detect 13 samples (three GN and nine GP) that were detected by BC processing and 16 samples were not identified at the species level. Other studies have also reported discordant results when comparing two or more methodologies for bacterial detection and identification from clinical samples [17]. These discrepancies may be caused by the presence of substances such as heme, IgG, sodium polyanethol sufonate or hemocomponents, in clinical samples or BACTEC® bottles [18, 19].

One false positive result was identified as GP and CoNS by qPCR assays with a Ct rate of 12,793. This patient gave a positive BC result with CoNS two days later, suggesting that the infection was also present in the previous sample but was not detectable by the phenotypic method. Similar results have been reported in other studies in which pathogens detected only by PCR were also present in other samples from the same patient that tested negative on phenotypic testing [20, 21].

The results obtained using the qPCR and phenotypic methods for determining methicillin susceptibility showed discrepancies in three *S. aureus* samples. One *S. aureus* sample was detected as methicillin susceptible, but the *mec*A gene was detected by qPCR. The other two samples were methicillin resistant, but the *mec*A gene was not detected in BC samples. When qPCR was performed directly from the clinical isolate strains and confirmatory tests for methicillin susceptibility were conducted, these three samples were determined to be methicillin resistant. These findings illustrated the fact that both phenotypic and molecular methods were not 100 % accurate at determining the susceptibility profile of clinical samples. The correlation between detection of methicillin resistance by the phenotypic method and detection of the *mec*A gene by SeptiFast® was evaluated in a recent study and the molecular method was unable to detect the *mec*A gene in two samples. The authors suggested that this was due to the difference in sensitivity between the two tests: one was conducted directly from clinical samples and the other directly from the isolates [22].

The phenotypic results of susceptibility tests of GN isolates compared with the qPCR results showed discrepancies in ESβL detection for six samples. In this study we did not evaluate the presence of the enzyme groups VEB, GES, PER, and OXA, which can eventually be found composing the same integron and are sporadically reported in some countries in South America [23–25].

Five carbapenem-resistant samples were detected in this study that were negative for the MβL-coding gene *bla*_*SPM*_, even when analyzing the clinical isolate. A Brazilian study reported a rate of 81.1 % carbapenem-resistant *P. aeruginosa* strains that were non-MβL producing [26]. Another study conducted at the same institution demonstrated that isolates of *P. aeruginosa* resistant to carbapenems that lacked MβL production had been detected in their hospital since 2000, and they proposed that other resistance mechanisms may be involved, such as changes in permeability through the membrane, porin loss or efflux systems [27].

The qPCR assay is not sufficiently comprehensive to detect all of the possible mechanisms of resistance expressed by microorganisms. However, because it is an “in-house” method, it is possible to customize the assay by adding or deleting genes to be tested depending on the particular resistance mechanisms of interest.

Our molecular method was able to detect the presence of fungi DNA in 100 % of samples determined to be positive by the phenotypic method (*n* = 14), however, it was only able to detect the specific species in three samples of *Candida* spp. It has previously been suggested that some positive samples may not be detected by commercial kits because the concentration of microorganisms in BC bottles is usually less than 100 CFU/mL [28].

Molecular analysis of whole blood directly was able to detect 12.1 % of GP/GN positive samples. The qPCR also detected one vancomycin-resistant *E. faecium* sample containing *van*A and 11 *mec*A positive samples, of which six gave the same result using the automated system. A study conducted with 23 episodes of febrile neutropenia in children detected the presence of bacterial DNA in three cases (13 %) by culture and in 11 cases (48 %) by PCR [29]. In another study conducted in Chile of children with cancer in which molecular detection of three species (*S. aureus*, *P. aeruginosa* and *E. coli*) directly from whole blood was performed, the authors reported a positivity rate of 20 % [7].

Some authors have reported higher sensitivity for detecting pathogens directly from whole blood [7, 30] and suggest that the volume of blood collected is highly dependent upon the weight of the patient. They concluded that it is necessary to collect up to 4.5 % of blood volume (about 4 ml/kg) to detect low concentrations of pathogens in blood [31]. The methodology applied in our study determined a volume of 1.5 ml of whole blood, regardless of patient weight. When considering only children and adolescents (as in our study), taking into account the weight of each patient when determining the volume of the sample to be collected would improve the sensitivity of the method.

## Conclusion

Real-time PCR applied directly to samples from positive bloodstream bottles or whole blood samples constitutes an accurate tool for the early identification of pathogens and antimicrobial resistance genes in bloodstream infections of pediatric oncologic patients. In addition to providing clinical data relevant to each individual patient, these molecular results would be invaluable in determining appropriate, patient-specific, antibiotic therapy.

## Abbreviations

PCR: Polymerase chain reaction
qPCR: Real-time polymerase chain reaction
BC: Bloodculture
BSI: Bloodstream infection
GP: Gram-positive
GN: Gram-negative
CoNS: Coagulase negative *Staphylococcus*
HFE: Hemochromatosis
MβL: Metallo-β-lactamase
Ct: Cycle Threshold value
ESβL: Extended-spectrum β-lactamase
MIC: Minimum inhibitory concentration
